# Supplementary material for: Single Cell Transcriptomic and Chromatin Profiles Suggest Layer Vb Is the Only Layer With Shared Excitatory Cell Types in the Medial and Lateral Entorhinal Cortex
Source: Front Neural Circuits. 2022 Jan 26;15:806154. doi: 10.3389/fncir.2021.806154 (PMC8826650; doi:10.3389/fncir.2021.806154)
Supplement: Supplementary file 14 [file Data_Sheet_1.ZIP › Inh_vs_Exc_Motifs/homerResults/motif1.similar.html]

motif1

## Information for motif1

G
A
C
T
C
T
A
G
A
G
T
C
C
G
T
A
A
C
T
G
A
G
T
C
A
C
G
T
A
C
T
G
A
T
C
G
T
G
C
A
  
Reverse Opposite:  

A
C
G
T
T
A
G
C
A
G
T
C
G
T
C
A
A
C
T
G
G
T
A
C
A
C
G
T
A
C
T
G
G
A
T
C
C
T
G
A
  

|  |  |
| --- | --- |
| p-value: | 1e-194 |
| log p-value: | -4.470e+02 |
| Information Content per bp: | 1.763 |
| Number of Target Sequences with motif | 4142.0 |
| Percentage of Target Sequences with motif | 33.83% |
| Number of Background Sequences with motif | 8518.6 |
| Percentage of Background Sequences with motif | 22.09% |
| Average Position of motif in Targets | 274.2 +/- 236.7bp |
| Average Position of motif in Background | 319.9 +/- 323.3bp |
| Strand Bias (log2 ratio + to - strand density) | -0.0 |
| Multiplicity (# of sites on avg that occur together) | 1.24 |
| Motif File: | file (matrix) reverse opposite |

### Similar de novo motifs found

|  |  |  |  |  |  |  |  |
| --- | --- | --- | --- | --- | --- | --- | --- |
| Rank | Match Score | Redundant Motif | P-value | log P-value | % of Targets | % of Background | Motif file |
| 1 | 0.961 | G T C A G T C A G A T C T C A G A G T C C G T A A C T G A G T C A C G T A C T G A G T C C T G A | 1e-189 | -436.853644 | 15.76% | 7.75% | motif file (matrix) |
| 2 | 0.955 | A G T C C G T A A C T G A G T C A C G T A C T G A G T C C T G A | 1e-184 | -423.946234 | 26.69% | 16.32% | motif file (matrix) |
| 3 | 0.779 | A C G T A T G C G T C A C A T G G T A C C A G T C T A G T C G A T C G A C G A T | 1e-44 | -101.871158 | 37.30% | 31.33% | motif file (matrix) |
| 4 | 0.644 | T C A G A T C G A T G C G T A C C A T G T A G C A C G T T C A G | 1e-31 | -71.385355 | 41.33% | 36.22% | motif file (matrix) |
| 5 | 0.792 | G T A C C G T A A G T C C G T A A C G T A G T C A C G T A C T G | 1e-28 | -64.494738 | 11.17% | 8.28% | motif file (matrix) |
| 6 | 0.618 | C G T A A G T C A C T G A C T G A G T C A C G T A C T G C T A G A G T C A G T C | 1e-6 | -16.039008 | 0.09% | 0.01% | motif file (matrix) |
| 7 | 0.744 | A G T C C G T A A C T G A G T C A C G T A C T G A C T G A C T G A C G T A C T G | 1e-1 | -3.037913 | 0.56% | 0.45% | motif file (matrix) |
| 8 | 0.689 | A C G T C G T A A G T C C G T A A C T G A G T C A C T G A C T G | 1e0 | -1.719820 | 0.41% | 0.36% | motif file (matrix) |
